# Supplementary material for: Massive image-based single-cell profiling reveals high levels of circulating platelet aggregates in patients with COVID-19
Source: Nat Commun. 2021 Dec 9;12:7135. doi: 10.1038/s41467-021-27378-2 (PMC8660840; doi:10.1038/s41467-021-27378-2)
Supplement: Supplementary file 1 — Supplementary Information [file 41467_2021_27378_MOESM1_ESM.pdf]

Supplementary Information

for

Massive image-based single-cell profiling reveals high levels of circulating platelet aggregates in patients with COVID-19

by Nishikawa et al.

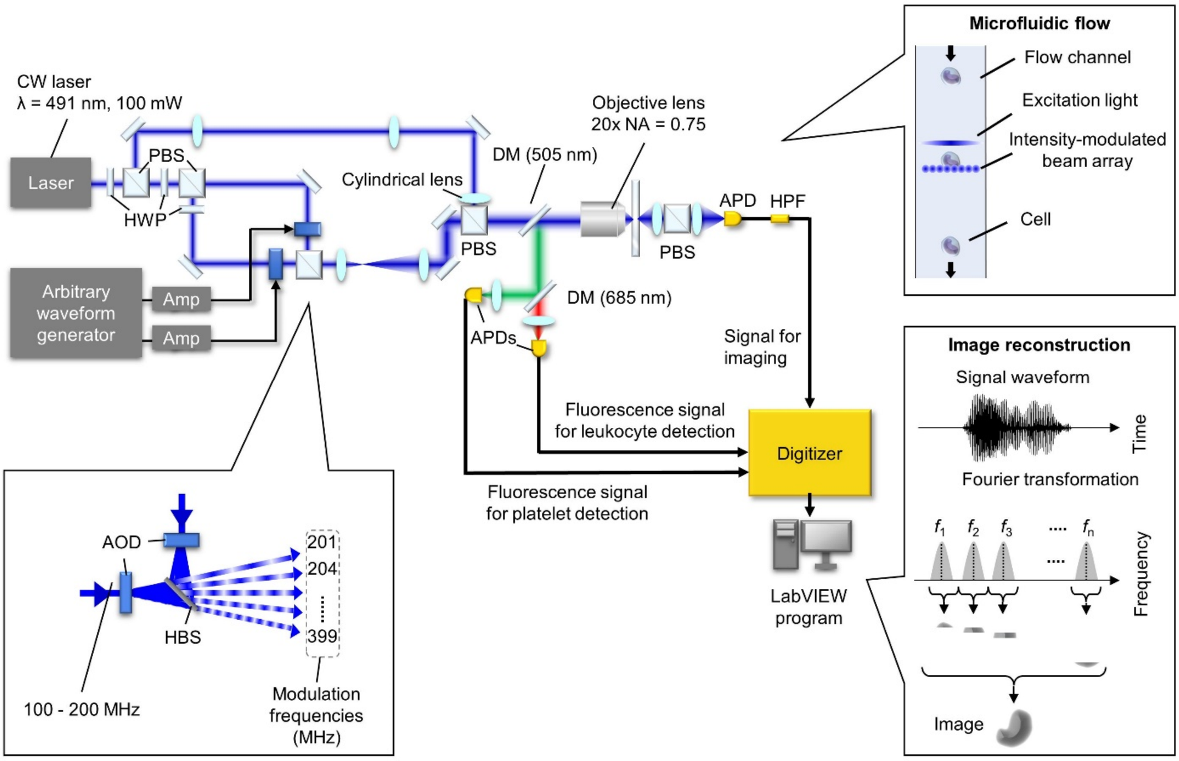

Supplementary Fig. 1 | Detailed schematic of the FDM microscope. CW: continuous wave; PBS: polarizing beamsplitter; HWP: half-wave plate; APD: avalanche photodetector; HPF: high-pass filter; DM: dichroic mirror; HBS: half beamsplitter; AOD: acousto-optic deflector; NA: numerical aperture. See “Optical frequency-division-multiplexed microscope” in the Methods section for details.

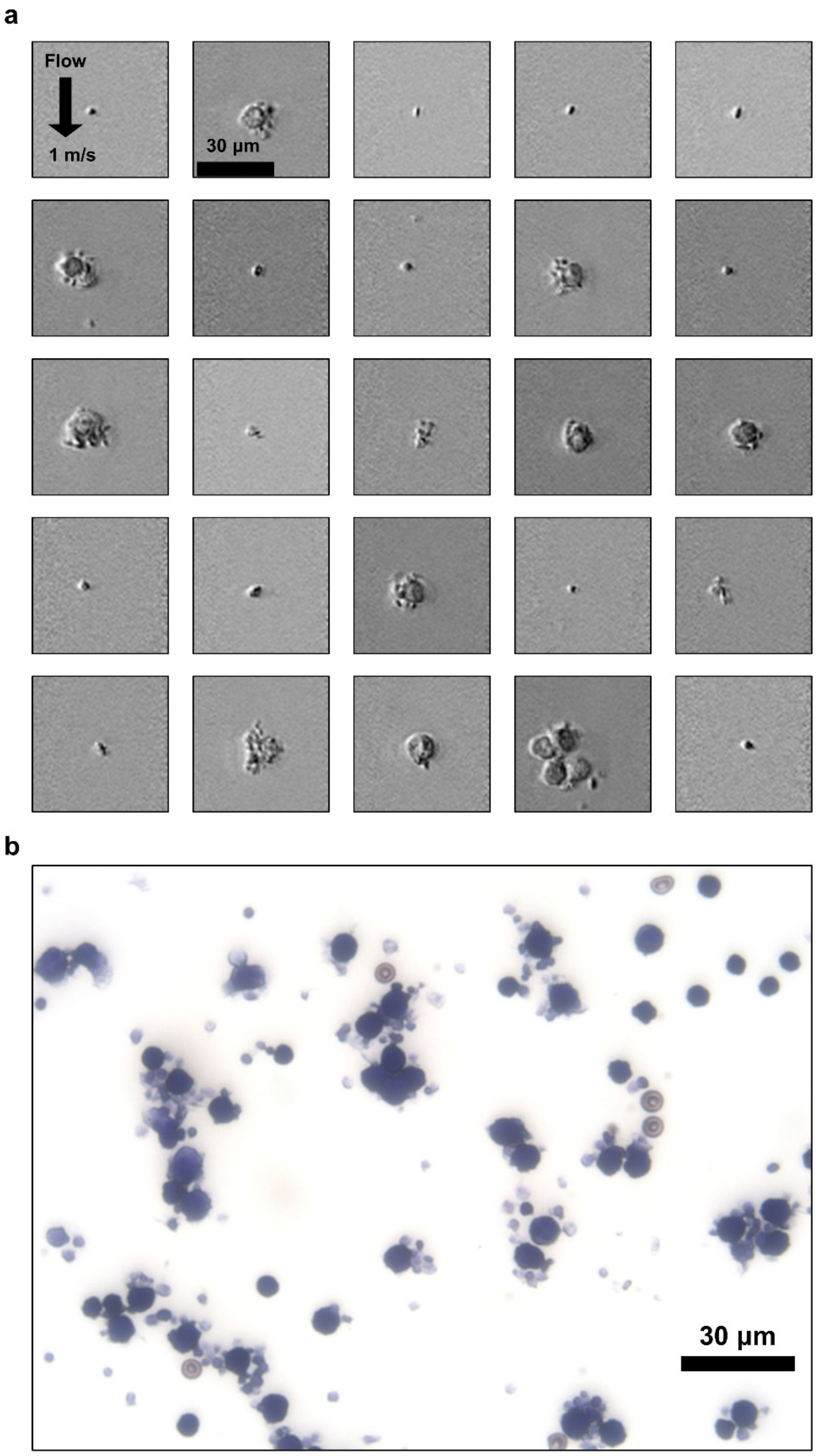

Supplementary Fig. 2 | Images of single platelets and platelet aggregates. a, Images of flowing single platelets and platelet aggregates obtained by the FDM microscope. **b,** Image of stationary single platelets, platelet aggregates, and leukocytes obtained by a conventional optical microscope. The sample was prepared using cytospin, followed by May-Giemsa staining. Both samples were obtained from a blood sample collected from a single COVID-19 patient on the same blood draw date.

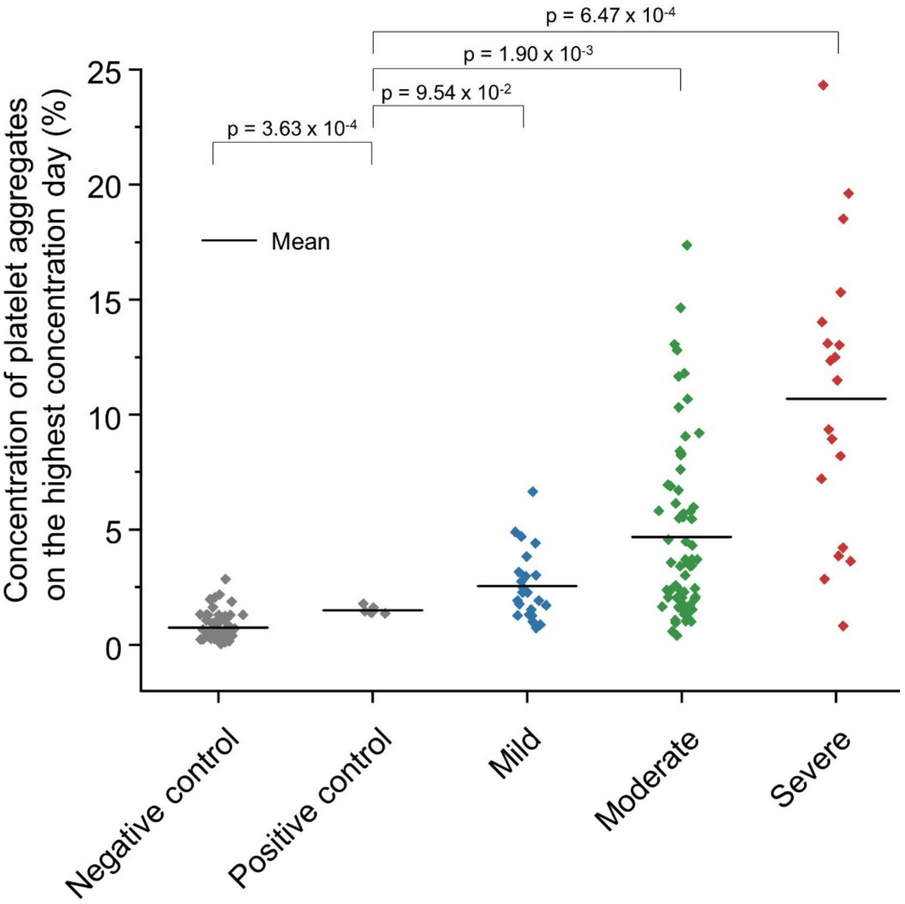

Supplementary Fig. 3 | Comparison with negative and positive control groups. Negative control: healthy subjects (n = 67 biologically independent samples); positive control (n = 7 biologically independent samples): patients under no anticoagulant therapy with no abnormality confirmed by their coagulation tests (see “human subjects” in the Methods section for details); Mild: mild COVID-19 patients (n = 23 biologically independent samples); Moderate: moderate COVID-19 patients (n = 68 biologically independent samples); Severe: severe COVID-19 patients (n = 19 biologically independent samples). Samples were measured on the highest concentration day of each hospitalized patient. Exact p values were obtained using the Mann–Whitney U-test (two-sided) and shown in the figure. Source data are provided as a Source Data file.

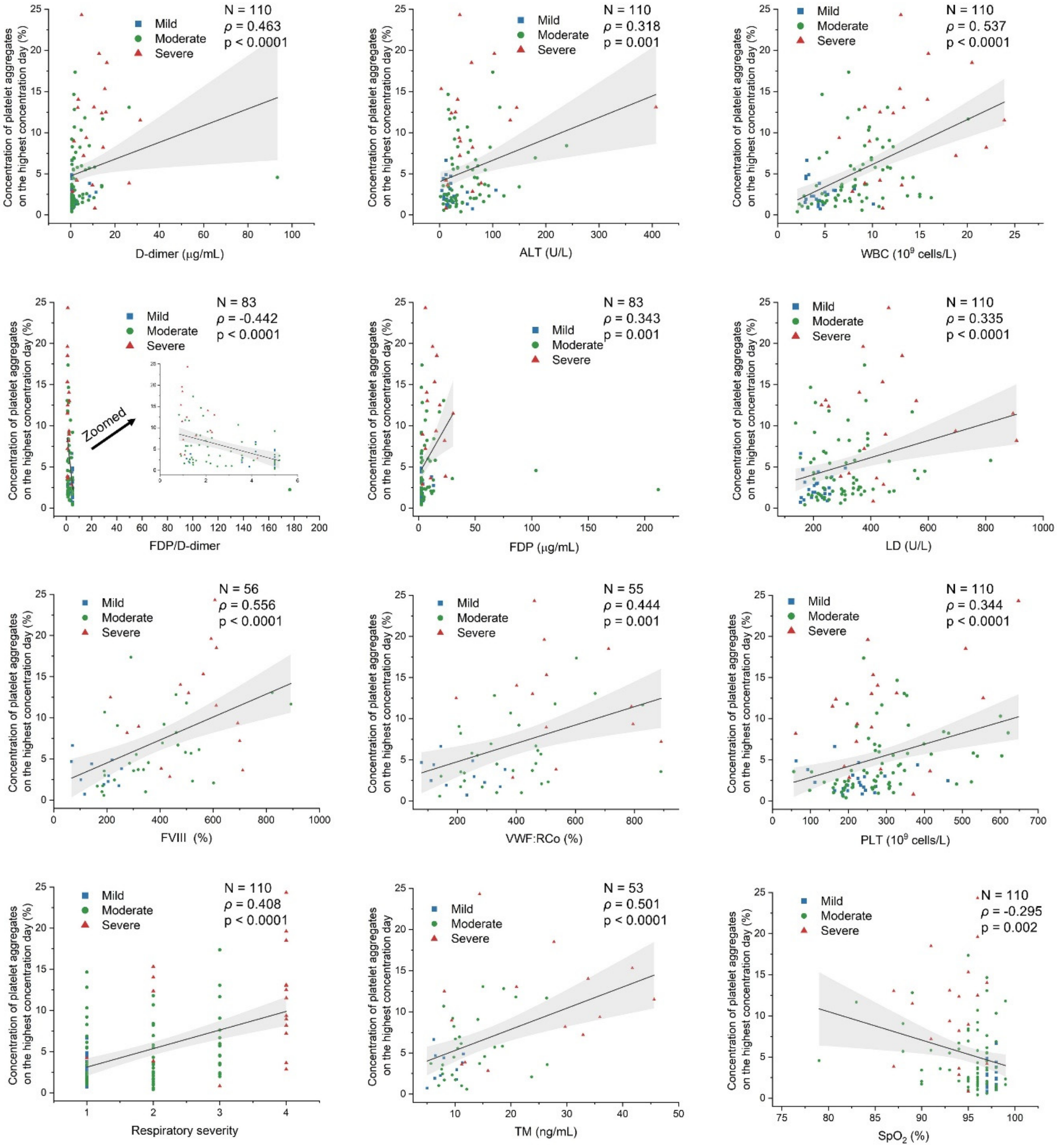

Supplementary Fig. 4 | Strong correlations between the concentration of platelet aggregates and clinical laboratory and physical findings. Respiratory severity level 1: without oxygen administration; level 2: with oxygen administration of 0.5 – 4 L/min; level 3: with oxygen administration of ≥ 5 L/min; level 4: with tracheal intubation or VV-ECMO. Linear fits show the correlation between the concentration of platelet aggregates and each clinical parameter with a 95% confidence interval shown in gray calculated by the standard error of measured y values. P values were obtained using the two-sided ANOVA test and shown in the figure. Source data are provided as a Source Data file.

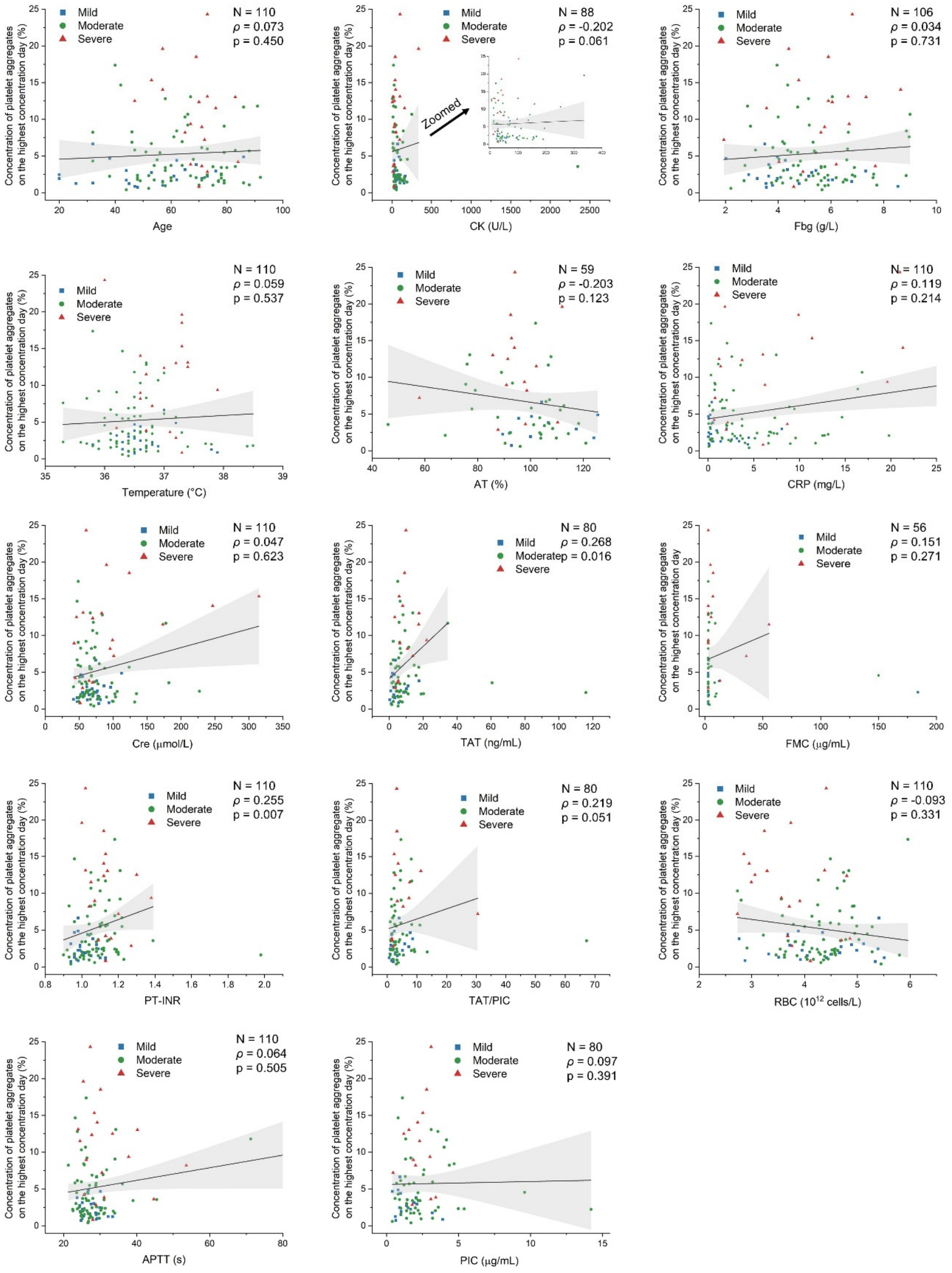

Supplementary Fig. 5 | Weak correlations between the concentration of platelet aggregates and clinical laboratory and physical findings. Linear fits show the correlation between the concentration of platelet aggregates and each clinical parameter with a 95% confidence interval shown in gray calculated by the standard error of measured y values. P values were obtained using the two-sided ANOVA test and shown in the figure. Source data are provided as a Source Data file.

Supplementary Data 1 | Demographics, clinical characteristics, and laboratory findings of patients with COVID-19. All the patients in this study were hospitalized at the University of Tokyo Hospital. Data are expressed as median values (IQR), n (%), or n/N (%). p values were calculated by the *t* test, Mann-Whitney U test, one-way ANOVA, chi-squared test, or Fisher's exact test.

	All patients	Mild patients	Moderate patients	Severe patients	p value	Survivors	Non-survivors	p value
	n = 110	n = 23	n = 68	n = 19		n = 99	n = 11	
Age, years	65.0 (52.0-73.5)	53.0 (36.0-65.0)	65.5 (53.3-78.0)	70.0 (65.0-73.0)	0.0004	62.0 (49.0-73.0)	83.0(72.0-88.0)	< 0.0001
Sex					0.077			0.75
Male	73 (66%)	11 (48%)	47 (69%)	15 (79%)		65 (66%)	8 (73%)	
Female	37 (34%)	12 (52%)	21 (31%)	4 (21%)		34 (34%)	3 (27%)	
Comorbidity								
Hypertension	55 (50%)	11 (48%)	33 (49%)	11 (58%)	0.75	49 (49%)	6 (55%)	0.95
Diabetes	35 (32%)	7 (30%)	19 (30%)	9 (47%)	0.27	31 (31%)	4 (36%)	0.74
Obesity, BMI > 25	38 (35%)	7 (30%)	27 (40%)	4 (21%)	0.29	36 (36%)	2 (18%)	0.32
COPD	4 (4%)	0	3 (4%)	1 (6%)	0.95	2 (2%)	2 (18%)	0.049
Coronary heart disease	6 (5%)	0	5 (7%)	1 (6%)	0.95	4 (4%)	2 (18%)	0.11
Chronic liver disease	9 (8%)	3 (13%)	4 (6%)	2 (12%)	0.95	8 (8%)	1 (9%)	0.95
Chronic kidney disease	9 (8%)	3 (13%)	4 (6%)	2 (11%)	0.95	7 (7%)	2 (18%)	0.22
Active malignancy	7 (6%)	0	6 (9%)	1 (6%)	0.95	5 (5%)	2 (18%)	0.14
Antiplatelet therapy at baseline	12 (11%)	3 (13%)	8 (12%)	1 (6%)	0.95	10 (10%)	2 (18%)	0.34
Anticoagulant therapy on the first measurement day	76 (69%)	13 (57%)	45 (66%)	18 (95%)	0.02	67 (68%)	9 (82%)	0.5
Laboratory findings on the first measurement day								
Leukocyte count, $\times 10^9$/L	6.4 (4.2-9.8)	4.0 (3.1–4.8)	6.9 (4.7-9.6)	11.1 (9.0-14.5)	< 0.0001	6.1 (4.2-9.7)	8.4 (7.0-10.7)	0.13
Red cell count, $\times 10^{12}$/L	4.25 (3.80-4.62)	4.34 (3.72-4.99)	4.29 (3.94-4.63)	4.11 (3.59-4.36)	0.084	4.27 (3.88-4.64)	4.07 (3.44-4.46)	0.042
Platelet count, $\times 10^9$/L	236.5 (181.8-299.3)	227.0 (175.0-245.0)	248.5 (183.0-312.0)	212.0 (180.0-328.0)	0.32	243.0 (186.0-308.0)	180.0 (132.0-209.0)	0.017
ALT, U/L	27.5 (14.0-49.8)	13.0 (12.0-27.0)	31.0 (17.0-51.3)	34.0 (14.0-105.0)	0.013	25.0 (14.0-49.0)	35.0 (17.0-92.0)	0.23
Creatinine, μmol/L	69.4 (58.1-80.7)	64.5 (54.8-78.7)	69.4 (61.0-81.8)	69.8 (59.2-115.8)	0.42	68.1 (57.5-79.6)	76.0 (69.0-155.6)	0.25
Lactate dehydrogenase, U/L	301.0 (220.8-379.5)	205.0 (169.0-240.0)	310.5 (238.3-359.0)	560.0 (384.0-660.0)	< 0.0001	290.0 (216.0-360.0)	564.0 (342.0-636.0)	< 0.0001
C-reactive protein, mg/L	4.15 (1.54-7.87)	0.32 (0.09-2.52)	5.24 (2.20-9.44)	6.66 (3.72-18.81)	< 0.0001	6.37 (3.02-20.56)	3.88 (1.25-7.50)	0.0077
PT-INR	1.05 (1.00-1.13)	0.99 (0.98-1.03)	1.07 (1.02-1.13)	1.13 (1.07-1.22)	0.36	1.05 (1.00-1.13)	1.06 (0.98-1.22)	0.46
APTT, s	27.6 (25.4-30.9)	26.8 (25.1-29.0)	27.2 (25.1-30.7)	29.5 (27.4-36.0)	0.0075	27.2 (25.1-29.8)	38.4 (32.3-45.5)	< 0.0001
D-dimer, μg/mL								
≤ 1	48 (44%)	19 (83%)	28 (41%)	1 (5%)	< 0.0001	47 (47%)	1 (9%)	0.022
> 1	62 (56%)	4 (17%)	40 (59%)	18 (95%)		52 (53%)	10 (91%)	
Anticoagulant therapy on the highest concentration day	84 (76%)	15 (65%)	51 (75%)	18 (95%)	0.074	76 (77%)	8 (73%)	0.72
Laboratory findings on the highest concentration day								
Leukocyte count, $\times 10^9$/L	8.0 (4.7-10.9)	4.3 (3.2-5.0)	8.2 (5.4-10.5)	12.9 (9.7-15.9)	< 0.0001	7.6 (4.5-10.1)	13.3 (8.2-20.1)	< 0.0001
Red cell count, $\times 10^{12}$/L	4.29 (3.72-4.68)	4.42 (3.72-4.99)	4.36 (3.98-4.68)	3.71 (3.06-4.38)	0.0027	4.36 (3.82-4.70)	3.64 (2.99-4.28)	0.0046
Platelet count, $\times 10^9$/L	263.0 (197.8-327.3)	227.0 (185.0-245.0)	276.0 (202.0-337.0)	261.0 (202.0-371.0)	0.046	269.0 (200.0-332.0)	221.0 (158.0-261.0)	0.019
ALT, U/L	31.0 (16.0-62.0)	16.0 (12.0-25.0)	33.5 (19.3-68.8)	38.0 (22.0-78.0)	0.017	30.0 (15.0-62.0)	38.0 (28.0-92.0)	0.19
Creatinine, μmol/L	68.5 (55.7-83.3)	69.0 (54.8-78.7)	68.1 (57.7-79.1)	69.8 (54.8-100.8)	0.32	67.2 (54.8-77.8)	100.8 (83.1-173.3)	0.12
Lactate dehydrogenase, U/L	280.0 (215.8-374.0)	210.0 (170.0-240.0)	301.0 (229.8-363.3)	420.0 (324.0-509.0)	< 0.0001	257.0 (211.0-351.0)	544.0 (389.0-695.0)	< 0.0001
C-reactive protein, mg/L	2.52 (0.63-6.96)	0.39 (0.09-2.57)	2.72 (1.05-7.27)	6.01 (1.83-19.62)	0.0004	2.23 (0.51-6.60)	9.45 (1.11-26.31)	< 0.0001
PT-INR	1.08 (1.01-1.13)	0.99 (0.98-1.06)	1.09 (1.03-1.15)	1.12 (1.05-1.16)	0.0059	1.06 (1.00-1.13)	1.14 (1.08-1.20)	0.017
APTT, s	27.2 (25.2-30.1)	26.8 (25.1-29.0)	26.8 (25.0-30.1)	28.8 (26.2-33.4)	0.44	26.8 (25.1-29.0)	37.8 (30.5-53.6)	< 0.0001
D-dimer, μg/mL								
≤ 1	43 (39%)	19 (83%)	23 (34%)	1 (5%)	< 0.0001	43 (43%)	0	0.0002
> 1	67 (61%)	4 (17%)	45 (66%)	18 (95%)		56 (57%)	11 (100%)	

Supplementary Data 2 | Measurement days of patients with COVID-19. Most patients were tested during their hospital stays while some patients were tested at the time of outpatient consultations after their discharge from the hospital.

Patient No.	Severity	Time after hospitalization (day)																																																		
		1	2	3	4	5	6	7	8	9	10	11	12	13	14	15	16	17	18	19	20	21	22	23	24	25	26	27	28	29	30	31	32	33	34	35	36	37	38	39	40	41	42	43	44	45	46	47	48	49	50		
1	severe																																																			
2	severe																																																			
3	moderate																																																		53	
4	mild																																																			
5	moderate																																																			
6	mild																																																		51	
7	moderate																																																		51	
8	mild																																																			
9	moderate																																																			
10	severe																																																		62	101
11	moderate																																																			
12	moderate																																																			
13	severe																																																			
14	moderate																																																			
15	severe																																																			
16	moderate																																																			
17	moderate																																																			
18	mild																																																			
19	mild																																																			
20	moderate																																																			
21	moderate																																																			
22	moderate																																																			
23	moderate																																																			
24	severe																																																			
25	severe																																																			
26	mild																																																			
27	moderate																																																			
28	moderate																																																			
29	severe																																																			
30	moderate																																																			
31	mild																																																			
32	moderate																																																			
33	moderate																																																			
34	severe																																																			
35	moderate																																																			
36	mild																																																			
37	moderate																																																			
38	severe																																																			
39	moderate																																																			
40	mild																																																			
41	moderate																																																			
42	mild																																																			
43	moderate																																																			
44	moderate																																																			
45	moderate																																																		62	
46	severe																																																			
47	moderate																																																			
48	moderate																																																			
49	mild																																																			
50	moderate																																																			
51	severe																																																			
52	moderate																																																			
53	mild																																																			
54	mild																																																			
55	mild																																																			
56	moderate																																																			
57	moderate																																																			
58	severe																																																			
59	moderate																																																			
60	moderate																																																			
61	moderate																																																			
62	mild																																																			
63	moderate																																																			
64	moderate																																																			
65	moderate																																																			
66	moderate																																																			
67	severe																																																		57	61
68	moderate																																																			
69	moderate																																																			
70	moderate																																																			
71	moderate																																																			
72	severe																																																			
73	moderate																																																			
74	moderate																																																			
75	moderate																																																			
76	moderate																																																			
77	moderate																																																			
78	severe																																																			
79	moderate																																																		54	
80	moderate																																																			
81	moderate																																																			
82	moderate																																																			
83	moderate																																																			
84	moderate																																																			
85	mild																																																			
86	severe																																																			
87	severe																																																			
88	moderate																																																			
89	moderate																																																			
90	mild																																																			
91	mild																																																			
92	mild																																																			
93	moderate																																																			
94	severe																																																			
95	moderate																																																			
96	moderate																																																			
97	moderate																																																			
98	moderate																																																			
99	moderate																																																			
100	moderate																																																			
101	moderate																																																			
102	moderate																																																			
103	mild																																																			
104	mild																																																			
105	moderate																																																			
106	moderate																																																			
107	moderate																																																			
108	moderate																																																			
109	mild																																																			
110	mild																																																			

- Measurement day during hospital stay
- Highest concentration day during hospital stay
- Outpatient consultation day after discharge from hospital
- Highest concentration day after discharge from hospital

Supplementary Data 3 | Additional laboratory findings of patients with COVID-19. All the patients in this study were hospitalized at the University of Tokyo Hospital. p values were calculated by *t* test, Mann-Whitney U test, one-way ANOVA, chi-squared test, or Fisher's exact test.

	All patients	Mild patients	Moderate patients	Severe patients	p value	Survivors	Non-survivors	p value
FVIII, %	329.2 (205.7-514.9) n = 56	189.8 (102.8-220.1) n = 11	342.4 (219.9-497.2) n = 30	563.1 (404.9-612.4) n = 15	< 0.0001	311.0 (191.1-464.7) n = 45	517.3 (301.1-692.7) n = 11	0.0013
TM, ng/mL	11.2 (8.1-18.6) n = 53	7.7 (6.4-10.6) n = 10	10.8 (8.8-16.2) n = 29	24.4 (11.7-34.3) n = 14	< 0.0001	10.2 (8.0-12.7) n = 42	26.4 (20.9-32.9) n = 11	< 0.0001
VWF:Rco, %	380.9 (224.1-494.5) n = 55	232.1 (120.2-292.9) n = 11	380.9 (224.1-483.5) n = 31	501.4 (427.9-751.2) n = 13	0.0001	333.5 (218.0-463.7) n = 45	660.6 (476.3-843.4) n = 10	< 0.0001
FDP, µg/mL	3.4 (2.5-10.9) n = 83	2.5 (2.5-3.5) n = 16	3.4 (2.5-9.2) n = 52	12.3 (5.9-18.6) n = 15	0.50	3.4 (2.5-8.3) n = 72	13.2 (7.1-22.9) n = 11	0.28
TAT, ng/mL	6.0 (3.5-10.2) n = 80	3.1 (1.5-7.9) n = 16	6.3 (4.3-10.4) n = 50	8.6 (5.3-14.8) n = 14	0.35	5.5 (3.1-8.9) n = 69	17.5 (10.8-22.1) n = 11	0.0031
FMC, µg/mL	3.0 (3.0-5.4) n = 55	3.0 (3.0-3.2) n = 11	3.0 (3.0-4.0) n = 31	4.8 (3.0-10.2) n = 13	0.70	3.0 (3.0-3.8) n = 45	6.1 (3.0-16.5) n = 10	0.20
PIC, µg/mL	1.9 (1.1-3.0) n = 80	1.0 (0.8-2.0) n = 16	1.9 (1.3-3.2) n = 50	2.2 (1.7-3.0) n = 14	0.16	1.9 (1.1-3.1) n = 69	1.6 (1.3-2.3) n = 11	0.25
Fbg, g/L	5.17 (4.03-6.20) n = 106	4.12 (3.55-5.55) n = 22	5.32 (4.19-6.33) n = 65	5.90 (4.59-6.69) n = 19	0.085	5.18 (4.17-6.20) n = 95	4.74 (2.95-5.63) n = 11	0.085
AT, %	100.0 (90.9-107.1) n = 59	100.4 (92.8-117.3) n = 11	103.5 (88.3-107.5) n = 33	93.9 (90.9-98.6) n = 15	0.19	101.6 (92.6-108.3) n = 48	77.8 (67.7-98.6) n = 11	< 0.0001
CK, U/L	47.0 (28.0-97.0) n = 87	56.5 (35.5-87.8) n = 16	50.0 (28.5-104.0) n = 53	38.0 (24.0-97.8) n = 18	0.72	47.0 (28.0-98.0) n = 77	46.5 (32.0-112.5) n = 10	0.0044

Supplementary Data 4 | Multivariate regression analysis. The upper part shows the first analysis while the lower part shows the second analysis, to determine explanatory factors for predicting the concentration of platelet aggregates on the highest concentration day.

Objective factor		Concentration of platelet aggregates on the highest concentration day	
Explanatory factor		Standardized coefficient (β)	p value
Vascular endothelial disorder markers	FVIII	0.568	< 0.001
	TM		NS
	VWF:Rco		NS
	R^2	0.322 (p < 0.001)	
Coagulation / fibrinolysis markers	PT-INR	0.222	0.049
	D-dimer		NS
	FDP		NS
	TAT		NS
	R^2	0.049 (p = 0.049)	
Other markers	WBC	0.265	0.009
	Respiratory severity	0.306	0.002
	PLT	0.227	0.006
	LD		NS
	ALT		NS
	SpO_2		NS
	Survival		NS
	Gender		NS
	R^2	0.364 (p < 0.001)	

Objective factor		Concentration of platelet aggregates on the highest concentration day	
Explanatory factor		Standardized coefficient (β)	p value
Selected markers	Respiratory severity	0.355	0.009
	FVIII	0.323	0.016
	WBC		NS
	PLT		NS
	PT-INR		NS
	R^2	0.352 (p < 0.001)	

Supplementary Data 5 | Demographics, clinical characteristics, and laboratory findings of patients with other diseases. All the patients in this study were hospitalized at the University of Tokyo Hospital. Data are expressed as median values (IQR) or n/N (%).

	Positive control
	n = 7
Age, years	47.0 (17.0-70.0)
Sex	
Male	3 (43%)
Female	4 (57%)
Comorbidity	
Hypertension	2 (29%)
Diabetes	1 (14%)
Obesity, BMI > 25	1 (14%)
Coronary heart disease	0
Active malignancy	0
Antiplatelet therapy	0
Anticoagulant therapy	0
Laboratory findings	
Leukocyte count, ×109/L	5.1 (4.7-5.2)
Platelet count, ×109/L	238.0 (218.5-283.0)
ALT, U/L	19.0 (15.5-29.0)
Creatinine, μmol/L	64.5 (59.7-74.7)
Lactate dehydrogenase, U/L	216.0 (196.0-223.0)
C-reactive protein, mg/L	0.02 (0.02-0.05)
D-dimer, μg/mL	0.5 (0.5-0.7)
