# Supplementary material for: Massive image-based single-cell profiling reveals high levels of circulating platelet aggregates in patients with COVID-19
Source: Nat Commun. 2021 Dec 9;12:7135. doi: 10.1038/s41467-021-27378-2 (PMC8660840; doi:10.1038/s41467-021-27378-2)
Supplement: Supplementary file 3 — Description of Additional Supplementary Files [file 41467_2021_27378_MOESM3_ESM.pdf]

Description of Additional Supplementary Files

File Name: Supplementary Data 1
Description: Demographics, clinical characteristics, and laboratory findings of patients with COVID-19.

File Name: Supplementary Data 2
Description: Measurement days of patients with COVID-19.

File Name: Supplementary Data 3
Description: Additional laboratory findings of patients with COVID-19.

File Name: Supplementary Data 4
Description: Multivariate regression analysis.

File Name: Supplementary Data 5
Description: Demographics, clinical characteristics, and laboratory findings of patients with other diseases.
