# Supplementary material for: Massive image-based single-cell profiling reveals high levels of circulating platelet aggregates in patients with COVID-19
Source: Nat Commun. 2021 Dec 9;12:7135. doi: 10.1038/s41467-021-27378-2 (PMC8660840; doi:10.1038/s41467-021-27378-2)
Supplement: Supplementary file 9 — Reporting Summary [file 41467_2021_27378_MOESM9_ESM.pdf]

nature portfolio

Corresponding author(s): Ting-Hui Xiao, Yutaka Yatomi, Keisuke Goda

Last updated by author(s): Oct 24, 2021

Reporting Summary

Nature Portfolio wishes to improve the reproducibility of the work that we publish. This form provides structure for consistency and transparency in reporting. For further information on Nature Portfolio policies, see our Editorial Policies and the Editorial Policy Checklist.

Statistics

For all statistical analyses, confirm that the following items are present in the figure legend, table legend, main text, or Methods section.

n/a	Confirmed	
☐	☒	The exact sample size (n) for each experimental group/condition, given as a discrete number and unit of measurement
☐	☒	A statement on whether measurements were taken from distinct samples or whether the same sample was measured repeatedly
☐	☒	The statistical test(s) used AND whether they are one- or two-sided<br>*Only common tests should be described solely by name; describe more complex techniques in the Methods section.*
☐	☒	A description of all covariates tested
☐	☒	A description of any assumptions or corrections, such as tests of normality and adjustment for multiple comparisons
☐	☒	A full description of the statistical parameters including central tendency (e.g. means) or other basic estimates (e.g. regression coefficient) AND variation (e.g. standard deviation) or associated estimates of uncertainty (e.g. confidence intervals)
☐	☒	For null hypothesis testing, the test statistic (e.g. F, t, r) with confidence intervals, effect sizes, degrees of freedom and P value noted<br>*Give P values as exact values whenever suitable.*
☒	☐	For Bayesian analysis, information on the choice of priors and Markov chain Monte Carlo settings
☐	☒	For hierarchical and complex designs, identification of the appropriate level for tests and full reporting of outcomes
☒	☐	Estimates of effect sizes (e.g. Cohen's d, Pearson's r), indicating how they were calculated

Our web collection on statistics for biologists contains articles on many of the points above.

Software and code

Policy information about availability of computer code

Data collection	LabVIEW2016 was used for our data collection. The program used for the data collection in this study is only available from the corresponding authors upon reasonable request since it requires specific measurement conditions (a proper digitizer, etc).
Data analysis	MATLAB R2020a was used for our image preprocessing, and Python 3 was used for our image analysis. In addition, Origin 2021b was used for our data analysis. All the codes used for the data analysis in this study are available on the Zenodo database under access code 5592561 [http://doi.org/10.5281/zenodo.5592561] and are also available from the corresponding authors upon reasonable request. SPSS software version 25 was used for our multivariate regression analysis.

For manuscripts utilizing custom algorithms or software that are central to the research but not yet described in published literature, software must be made available to editors and reviewers. We strongly encourage code deposition in a community repository (e.g. GitHub). See the Nature Portfolio guidelines for submitting code & software for further information.

Data

Policy information about availability of data

All manuscripts must include a data availability statement. This statement should provide the following information, where applicable:

- Accession codes, unique identifiers, or web links for publicly available datasets
- A description of any restrictions on data availability
- For clinical datasets or third party data, please ensure that the statement adheres to our policy

The source data (Source Data 1) used in this study are available on the Zenodo database with access code 5592602 [http://doi.org/10.5281/zenodo.5592602] and are also available from the corresponding authors upon reasonable request.

Field-specific reporting

Please select the one below that is the best fit for your research. If you are not sure, read the appropriate sections before making your selection.

☒ Life sciences ☐ Behavioural & social sciences ☐ Ecological, evolutionary & environmental sciences

For a reference copy of the document with all sections, see nature.com/documents/nr-reporting-summary-flat.pdf

Life sciences study design

All studies must disclose on these points even when the disclosure is negative.

Sample size	The sample size is determined by the number of the cellular images acquired. It is typically 25,000's for each blood sample. The blood samples were obtained from 110 COVID-19 patients, 4 healthy subjects, and 7 patients under no anticoagulant therapy with no abnormality confirmed by their coagulation tests.
Data exclusions	Non-cell images and low-quality (out-of-focus blurry) images were excluded from the analysis.
Replication	The data reproducibility was checked using blood samples from healthy subjects multiple times before testing blood samples from patients. Blood samples from a total of 110 COVID-19 patients (composed of 23 mild patients, 68 moderate patients, and 19 severe patients) were analyzed for high statistical accuracy. Image acquisition was performed at a frequency of 3-5 times per week per hospitalized patient, and the data were compared with negative control data, which were obtained from healthy subjects under the same sample preparation and image acquisition conditions on the same day to mitigate potential bias in the image data that may have come from experimental variations (e.g., fluctuations in optical alignment, hydrodynamic focusing conditions, blood draw, and sample preparation) and are shown in data presentation plots as references. Around 25,000's cellular images for each blood sample were measured to ensure high statistical accuracy.
Randomization	The COVID-19 subjects were categorized into three groups: (1) mild patient group: those requiring no oxygen therapy; (2) moderate patient group: those requiring oxygen therapy without mechanical ventilation for respiratory support such as extracorporeal membrane oxygenation (ECMO); (3) severe patient group: those requiring mechanical ventilation for respiratory support. In addition, 4 healthy subjects and 7 subjects under no anticoagulant therapy with no abnormality confirmed by their coagulation tests were used for comparison.
Blinding	The investigators were not blinded during the data collection and analysis since this study required clinical investigation.

Reporting for specific materials, systems and methods

We require information from authors about some types of materials, experimental systems and methods used in many studies. Here, indicate whether each material, system or method listed is relevant to your study. If you are not sure if a list item applies to your research, read the appropriate section before selecting a response.

Materials & experimental systems

n/a	Involved in the study	
☐	☒	Antibodies
☒	☐	Eukaryotic cell lines
☒	☐	Palaeontology and archaeology
☒	☐	Animals and other organisms
☐	☒	Human research participants
☒	☐	Clinical data
☒	☐	Dual use research of concern

Methods

n/a	Involved in the study	
☒	☐	ChIP-seq
☒	☐	Flow cytometry
☒	☐	MRI-based neuroimaging

Antibodies

Antibodies used	anti-CD61-PE (Beckman Coulter, IM3605)<br>anti-CD45-PC7 (Beckman Coulter, IM3548)
Validation	The above-listed antibodies are commercially available and have been validated by Beckman Coulter. Validation statements can be found on the websites of the manufacturer.

Human research participants

Policy information about [studies involving human research participants]()

Population characteristics	73 male subjects with COVID-19, 37 female subjects with COVID-19. Mean age: 65.0. More details can be found in Supplementary Table 1 and Supplementary Table 3. For other groups, see Supplementary Table 5.

Recruitment	All the subjects were hospitalized patients at the University of Tokyo Hospital alone and we did not do recruitment of participants outside the University of Tokyo Hospital. This may have introduced a selection bias, making it difficult to draw more general conclusions. Informed consent for participation in the study was obtained from the patients using an opt-out process on the webpage of the University of Tokyo Hospital. Patients who refused participation in our study were excluded. Written informed consent was obtained from the healthy subjects as well.
Ethics oversight	This study was conducted with the approval of the Institutional Ethics Committee in the School of Medicine at the University of Tokyo [no. 11049, no. 11344] in compliance with the relevant guidelines and regulations.

Note that full information on the approval of the study protocol must also be provided in the manuscript.
